# Therapeutic effects of Huangqi formula (Eefooton) in chronic kidney disease: clinical research and narrative literature review

**DOI:** 10.18632/aging.206170

**Published:** 2024-12-07

**Authors:** Kuo-Cheng Lu, San-Chiang Wu, Tsuo-Cheng Lu, I-Shang Tzeng, Chun-En Kuo, Yu-Chiang Hung, Szu-Ying Wu, Te-Chuan Chen, Ming-Kai Tsai, Chih-Kuang Chuang, Wen-Long Hu

**Affiliations:** 1 Division of Nephrology, Department of Medicine, Taipei Tzu Chi Hospital, Buddhist Tzu Chi Medical Foundation, New Taipei City 23142, Taiwan; 2 Division of Nephrology, Department of Medicine, Fu-Jen Catholic University Hospital, School of Medicine, Fu-Jen Catholic University, New Taipei City 24352, Taiwan; 3 Wu San-Chiang Medical Clinic, Lingya District, Kaohsiung City 802014, Taiwan; 4 Division of Nephrology, Department of Internal Medicine, Kaohsiung Armed Forces General Hospital, Lingya District, Kaohsiung City 80284, Taiwan; 5 Department of Chinese Medicine, Kaohsiung Chang Gung Memorial Hospital and Chang Gung University College of Medicine, Niaosong District, Kaohsiung 833, Taiwan; 6 Department of Statistics, School of Business, National Taipei University, New Taipei, Taiwan; 7 School of Chinese Medicine for Post-Baccalaureate, I-Shou University, Dashu District, Kaohsiung 840, Taiwan; 8 Department of Chinese Medicine, National Yang Ming Chiao Tung University, Beitou District, Taipei 112304, Taiwan; 9 Institute of Traditional Medicine, National Yang Ming Chiao Tung University, Beitou District, Taipei 112304, Taiwan; 10 Department of Chinese Medicine, Taipei City Hospital, Linsen, Chinese Medicine, and Kunming Branch, Datong District, Taipei 103212, Taiwan; 11 Division of Nephrology, Kaohsiung Chang Gung Memorial Hospital and Chang Gung University College of Medicine, Kaohsiung, Taiwan, Niaosong District, Kaohsiung 833, Taiwan; 12 Division of Nephrology, Chong Guang Hospital, Miaoli, Taiwan, Toufen City, Miaoli County 351, Taiwan; 13 Kaohsiung Medical University College of Medicine, Kaohsiung, Taiwan, Shihcyuan, Sanmin District, Kaohsiung 807, Taiwan; 14 Fooyin University College of Nursing, Kaohsiung, Taiwan, Ta-liao District, Kaohsiung 831, Taiwan

**Keywords:** chronic kidney disease, eefooton, Huangqi formula, herbal medicine, traditional chinese medicine

## Abstract

Objective: The study aimed to assess the clinical effects of employing the Huangqi formula (Eefooton; EFT) for chronic kidney disease (CKD) treatment. A narrative literature review was undertaken to elucidate the specific ingredients of EFT and their potential impact on renal health.

Methods: A retrospective observational study investigated EFT treatment in outpatients with stable CKD (stages 3B to 5) from March 2019 to March 2021. Patients received 20 mL of EFT thrice daily for 6 months, along with standard treatment. Control groups were matched to the EFT cohort. Regular assessments of renal, liver functions, and lipid profiles were conducted.

Results: Serum creatinine (Cr) and eGFR levels consistently improved in stage 3B CKD patients at each follow-up visit. At 6 months, improvement in Cr and eGFR was observed for stage 4 and 5 CKD. Stage 3B CKD patients exhibited notable reductions in systolic blood pressure after 3 and 6 months of EFT treatment. Remarkably, a substantial decrease in HbA1C was noted in stage 4 CKD individuals after three months of therapy. Additionally, stage 4 CKD patients saw a significant reduction in LDL levels after both 3 and 6 months of EFT treatment. A literature review on EFT ingredients indicated that the positive effects of EFT might be associated with its anti-inflammatory, antioxidant, and anti-fibrotic properties.

Conclusions: This research demonstrated that incorporating EFT alongside standard treatment enhanced renal function in individuals with CKD. EFT is proposed as a feasible complementary treatment for CKD patients, emphasizing the importance of early intervention.

## INTRODUCTION

Chronic kidney disease (CKD), marked by persistent indicators of kidney damage or reduced glomerular filtration rate (GFR) lasting more than 3 months, is now a major global public health concern [1]. Kidney function is intricately linked to various health complications such as malnutrition, anemia, hypertension, and bone disease, contributing to an overall decline in quality of life [2]. Effectively managing CKD is intricate and may entail patient discomfort, including potential medication side effects, lifestyle modifications, self-care management, and associated medical expenses [3].

CKD arises from intricate interactions involving inflammation, oxidative stress, and fibrosis, influencing both its initiation and advancement. Inflammation, the immune system’s response to infections or injuries [4], plays a crucial role in driving CKD development. Imbalances in pro- and anti-inflammatory markers escalate low-grade inflammation, correlating with increased mortality and cardiovascular complications [4, 5]. Factors like aging, diabetes, chronic inflammation, and uremic toxins contribute to heightened oxidative stress, significantly elevating CKD risk [6]. Kidney disease-related oxidative damage results from diminished antioxidants and heightened reactive oxygen species (ROS) production. The kidney’s heightened metabolic activity, rich in mitochondrial oxidation reactions, makes it susceptible to oxidative stress [7]. The research underscores the impact of oxidative stress in accelerating CKD progression, linking it to complications like hypertension, atherosclerosis, inflammation, and anemia [6, 8]. In CKD, the reciprocal cycle between oxidative stress and inflammation creates a dynamic interplay, with each factor magnifying the influence of the other [9]. Inflammatory processes stimulate ROS production, perpetuating oxidative stress, and further intensifying inflammation [10]. Tubulointerstitial fibrosis, a persistent and advancing condition affecting kidneys in aging and CKD, currently lacks specific treatment options. Recent breakthroughs have unveiled the cellular and molecular mechanisms driving renal fibrosis [11]. A notable aspect of the progression of CKD involves the deposition of extracellular matrix, chronic inflammation, tubule atrophy, fibrogenesis, and vascular rarefaction [12]. Recognizing this intricate relationship is crucial for developing targeted therapeutic interventions to mitigate CKD advancement.

Conventional interventions, including RAS blockers and SGLT2 inhibitors, aim to delay CKD progression [13–15]. However, using ACEIs or ARBs in older individuals and severe CKD is restricted due to potential risks like hyperkalemia and acute kidney injury [16]. The efficacy of traditional medical treatments for CKD is presently constrained, prompting an increasing interest in investigating complementary and alternative medicine (CAM) for the management of CKD [17]. Traditional Chinese medicine (TCM) is an economical and widely adopted complementary and alternative medicine (CAM) with a long history, especially prevalent in Asia. Numerous clinical studies have shown that TCM effectively manages early-stage CKD, leading to a significant decrease in the risk of progressing to end-stage renal disease (ESRD) [18–20]. Notably, the herbal formula Eefooton (EFT), part of TCM, has shown efficacy in slowing CKD progression. Comprising herbal extracts like *Astragalus membranaceus (A. membranaceus)* and *Rhodiola sacra (R. sacra)*, EFT demonstrates varied biological effects, including immunomodulatory properties, anti-oxidative stress, anti-inflammatory, and anti-fibrosis [21–23]. This study aims to clarify the clinical effects of EFT in individuals with CKD and includes a literature review on the potential positive effects of its components.

## RESULTS

Table 1 outlines the baseline demographic data of patients in the EFT group, categorized by their CKD stage. During the treatment period, four patients reported adverse events associated with EFT administration. Among these, one patient experienced parosmia, perceiving the smell of wood, while the others reported mild itching, and one patient presented with a skin rash. Notably, these adverse events were resolved without the need for additional treatment or discontinuation of EFT. In Table 2, demographic information and sequential changes in both eGFR and serum Cr levels within the control group are presented. No notable changes in eGFR and serum Cr levels were noted in the control group across all stages of CKD during the follow-up period.

**Table 1:** Baseline demographic data of patients in the EFT group, organized according to their chronic kidney disease (CKD) stage.

**Characteristics**	**Stage 3B CKD (n=43)**	**Stage 4 CKD (n=29)**	**Stage 5 CKD (n=16)**
**Age, years**	64.65 (9.57)	64.97 (13.96)	63.88 (7.90)
**Female, n (%)**	14 (32.56%)	12 (41.38%)	7 (43.75%)
**BMI, kg/m^2^**	24.54 (2.99)	24.13 (3.78)	24.85 (2.69)
**Systolic BP, mmHg**	141.69 (12.73)	139.57 (14.86)	141.44 (16.33)
**Diastolic BP, mmHg**	77.34 (9.63)	80.39 (11.83)	77.19 (7.17)
**eGFR, mL/min/1.73 m^2^**	37.55 (4.54)	22.01 (4.19)	9.42 (2.74)
**HbA_1c_, %**	7.21 (1.43)	7.37 (1.73)	6.25 (0.19)
**Hb, g/dL**	12.37 (1.59)	10.71 (1.86)	9.91 (1.70)
**Potassium, mg/dL**	4.38 (0.48)	4.51 (0.64)	4.85 (0.68)
**LDL, mg/dL**	90.56 (32.39)	88.00 (35.06)	73.55 (36.38)
**Type 2 DM, n (%)**	14 (32.56%)	8 (27.59%)	6 (37.50%)
**Hypertension, n (%)**	39 (90.70%)	28 (96.55%)	13 (81.25%)
**Medication, n (%)**			
**ACEi/ ARB**	32 (74.42%)	19 (65.52%)	11 (68.75%)
**CCB**	29 (67.44%)	17 (58.62%)	11 (68.75%)
**Sulfonylurea**	9 (20.93%)	4 (13.79%)	6 (37.50%)
**DPP-4i**	6 (13.95%)	4 (13.79%)	6 (37.50%)
**Insulin**	3 (6.98%)	2 (6.90%)	4 (25.00%)
**Statin**	32 (74.42%)	18 (62.07%)	11 (68.75%)

Note: All data is expressed as the mean (standard deviation).

Abbreviations: ACEi, angiotensin-converting enzyme inhibitor; ARB, angiotensin receptor blocker; BMI: body mass index; BP, blood pressure; CCB, calcium-channel blocker; CKD, chronic kidney disease; DM, diabetes mellitus; DPP-4i, dipeptidyl peptidase 4 inhibitors; eGFR, estimated glomerular filtration rate; Hb, hemoglobin.

**Table 2:** The demographic information and sequential alterations in both eGFR and serum Cr levels within the control group.

**Characteristics**	**Stage 3B CKD (n=44)**	**Stage 4 CKD (n=29)**	**Stage 5 CKD (n=16)**
Age, years	65.36(9.41)	66.41(14.93)	67.12(8.64)
Female, n (%)	14(31.82%)	12(41.38%)	7(43.75%)
BMI, kg/m^2^	26.17(5.76)	25.55(3.97)	24.36(4.83)
Systolic BP, mmHg	127.45(14.05)	135.06(19.13)	142.62(17.23)
Diastolic BP, mmHg	70.95(8.96)	71.82(10.48)	75.50(12.18)
**eGFR,** mL/min/1.73 m^2^			
baseline	40.82(6.04)	23.27(4.59)	9.09(2.91)
3 months	40.50(6.58)	24.54 (6.41)	9.52(3.41)
6 months	41.80(8.67)	24.06(7.79)	9.06(3.31)
**Cr,** mg/dL			
baseline	1.68(0.25)	2.73(0.62)	6.06(1.93)
3 months	1.71(0.31)	2.73(0.82)	5.89(2.06)
6 months	1.67(0.32)	2.78(0.80)	6.21(2.20)

Note: All data is expressed as the mean (standard deviation).

Abbreviations: BMI, body mass index; BP, blood pressure; Cr, Creatinine; eGFR, estimated glomerular filtration rate.

### Primary outcomes

#### Change of eGFR and serum Cr levels

The baseline, 3-month, and 6-month eGFR levels for the EFT study group were 27.31±11.70, 35.06±16.88, and 36.62±16.07 mL/min/1.73 m^2^, respectively. Notably, in stage 3B, a significant increase in eGFR was observed at both the 3-month and 6-month marks during the EFT treatment, compared to controls (all p<0.001). Furthermore, there was a significant improvement in GFR at 6 months for both the Stage 4 CKD and Stage 5 CKD groups (P=0.02 and P=0.01, respectively; Table 3). The EFT study group displayed Cr levels of 2.96±1.86, 2.48±1.59, and 2.22±1.33 mg/dL at baseline, 3-month, and 6-month treatment, respectively. However, a noteworthy decrease in Cr levels was observed in stage 3B at both the 3-month and 6-month EFT treatment intervals compared to controls (P<0.01 and P<0.001, respectively). Similarly, the stage 4 CKD and stage 5 CKD groups exhibited a significant decrease in Cr levels up to 6 months of EFT treatment (P=0.04 and P=0.03, respectively) (Table 3).

**Table 3:** The eGFR and Cr levels were compared at baseline, 3 months, and 6 months of treatment between the EFT treatment group and the control group.

**Characteristics**	**Baseline**			**3 months of treatment**			**6 months of treatment**		
**CKD stage**	**3B**	**4**	**5**	**3B**	**4**	**5**	**3B**	**4**	**5**
**EFT;** eGFR, mL/min/1.73 m^2^	37.55 (4.54)	22.01 (4.19)	9.42 (2.74)	47.93 *** (11.64)	28.17 (9.04)	12.08 (4.28)	50.31*** (7.41)	29.61* (10.85)	13.64 * (5.75)^*^
**Control;** eGFR, mL/min/1.73 m^2^	40.82 (6.04)	23.27 (4.59)	9.09 (2.91)	40.50 (6.58)	24.54 (6.41)	9.52 (3.41)	41.80 (8.67)	24.06 (7.79)	9.06 (3.31)
**EFT** vs. **Control**				**P<0.001**	**P= 0.08**	**P=0.07**	**P<0.001**	**P<0.05**	**P<0.05**
**EFT;** Cr, mg/dL	1.80 (0.26)	2.91 (0.64)	6.20 (2.01)	1.50** (0.30)	2.44 (0.73)	5.20 (1.67)	1.42*** (0.19)	2.38* (0.71)	4.52* (2.00)
**Control;** Cr, mg/dL	1.68 (0.25)	2.73 (0.62)	6.06 (1.93)	1.71 (0.31)	2.73 (0.82)	5.89 (2.06)	1.67 (0.32)	2.78 (0.80)	6.21 (2.20)
**EFT** vs. **Control**				**P<0.001**	**P=0.16**	**P=0.31**	**P=3.10**	**P=0.06**	**P<0.05**

Note: *P<0.05; **P<0.01; ***P<0.001 when compared with the baseline data.

**Figure 1:**
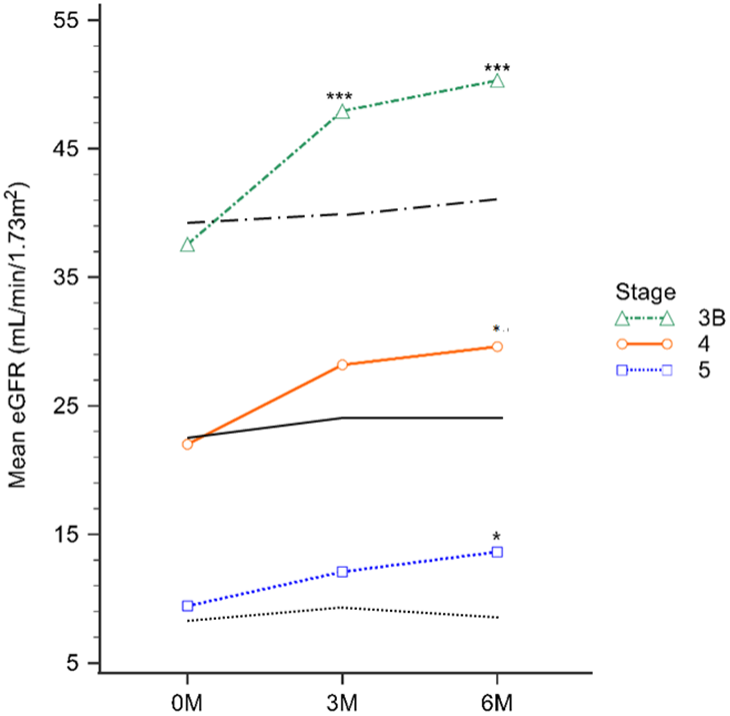
**The eGFR levels were evaluated across different CKD stages after 3 and 6 months of treatment.** Specifically, the green, red, and blue lines depict the EFT-treated CKD stage 3b, 4, and 5 groups, respectively, while the black line corresponds to the control group at the respective CKD stage. ^*^ P<0.05, ^***^ P<0.001.

**Figure 2:**
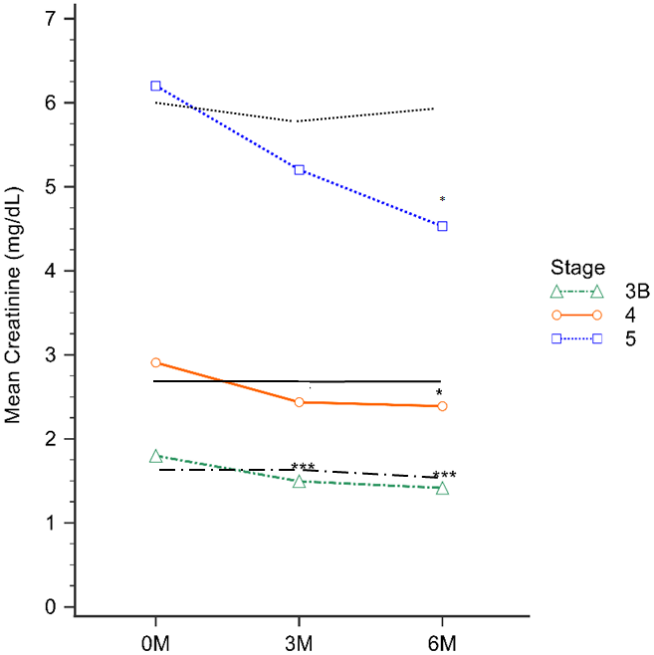
**The analysis involved assessing serum Cr levels in distinct CKD stages after 3 and 6 months of treatment.** Notably, the green, red, and blue lines represent the EFT-treated CKD stage 3b, 4, and 5 groups, while the black line corresponds to the control group within their respective CKD stages. ^*^ P<0.05, ^***^ P<0.001.

While there were no notable variances in baseline eGFR and serum Cr levels between the EFT and control groups, significant differences emerged in CKD stage 3b patients who were treated with EFT. Specifically, the EFT treatment group exhibited significantly higher eGFR (Figure 1) and lower Cr (Figure 2) levels after both 3 and 6 months of treatment (p<0.01, as indicated in Table 3**)**. In contrast, for CKD stages 4 and 5, the EFT treatment group demonstrated elevated eGFR and reduced Cr levels only after 6 months of treatment, in comparison to the control group (p<0.05, as indicated in Table 3 and Figures 1, 2).

### Secondary outcomes in the EFT treatment group

Significant reductions in systolic blood pressure (SBP) were observed within the stage 3B CKD cohort after 3 and 6 months of EFT treatment (P=0.002, P=0.019, respectively). Notably, a decrease in diastolic blood pressure (DBP) was evident among stage 4 CKD patients following three months of EFT therapy (P=0.046). Serum potassium levels showed no significant differences between CKD groups during each follow-up visit. A considerable reduction in HbA1C was noted in individuals diagnosed with stage 4 CKD after a three-month therapy period (P=0.040). Similarly, a slight decrease in Hb levels was observed among stage 4 CKD individuals after three months of EFT therapy (P=0.021). Furthermore, stage 4 CKD patients experienced a significant decrease in low-density lipoprotein (LDL) levels after both 3 and 6 months of EFT treatment (P=0.004 and P=0.001, respectively). Regarding liver enzyme levels, individuals with stage 3B CKD showed a significant decrease in alanine aminotransferase (GPT) levels after three and six months of EFT therapy (P=0.005 and P=0.032, respectively). Additionally, patients with stage 5 CKD exhibited a notable decrease in GPT levels after 6 months of EFT treatment (P=0.036, Table 4).

**Table 4:** Secondary outcome measurements were recorded at each visit based on the CKD stage within the EFT treatment group.

**Characteristics**	**Baseline**			**3 months of treatment**			**6 months of treatment**		
**CKD stage**	**3B**	**4**	**5**	**3B**	**4**	**5**	**3B**	**4**	**5**
Systolic BP, mmHg	141.69 (12.73)	139.57 (14.86)	141.44 (16.33)	134.71 (13.38)^**^	136.61 (17.96)	136.25 (14.94)	135.52 (12.71)^*^	139.21 (15.17)	139.71 (6.264)
Diastolic BP, mmHg	77.34 (9.63)	80.39 (11.83)	77.19 (7.17)	75.39 (9.33)	76.00 (8.70)^*^	75.00 (10.48)	78.43 (6.35)	79.37 (10.63)	75.29 (5.68)
Potassium, mg/dL	4.38 (0.48)	4.51 (0.64)	4.85 (0.68)	4.43 (0.52)	4.52 (0.47)	4.71 (0.77)	4.43 (0.39)	4.59 (0.53)	4.49 (0.62)
Hb, g/dL	12.37 (1.59)	10.71 (1.86)	9.91 (1.70)	12.18 (1.74)	10.22 (1.65)^*^	10.41 (1.27)	12.82 (1.94)	10.22 (1.79)	9.68 (1.18)
HbA_1c_, %	7.21 (1.43)	7.37 (1.73)	6.25 (0.19)	7.37 (1.09)	6.10 (0.57)^*^	-	6.95 (1.50)	-	^-^
LDL, mg/dL	90.56 (32.39)	88.00 (35.06)	73.55 (36.38)	93.05 (30.74)	66.37 (28.11)^**^	67.43 (18.51)	80.85 (29.19)	60.60 (15.50)^**^	81.00 (18.18)
GOT, U/L	22.33 (6.97)	23.88 (19.37)	20.50 (12.99)	21.80 (9.44)	21.09 (7.54)	16.80 (6.66)	20.73 (8.37)	19.86 (6.44)	15.20 (4.32)
GPT, U/L	22.81 (11.86)	28.16 (49.29)	21.75 (22.12)	18.54 (7.21)^**^	19.26 (16.79)	13.30 (7.41)	18.31 (7.98)^*^	11.71 (4.79)	10.40 (1.14)^*^

Note: All data is expressed as the mean (standard deviation).

Abbreviations: BP, blood pressure; CKD, chronic kidney disease; eGFR, estimated glomerular filtration rate; GOT, aspartate aminotransferase; GPT, alanine aminotransferase; Hb, hemoglobin; LDL, low-density lipoprotein. ^*^ *p*<0.05, ^**^ *p*<0.01, ^***^ *p*<0.001.

## DISCUSSION

In our retrospective observational study, EFT treatment led to significant improvements in eGFR and Cr levels for patients with stage 3B at each follow-up visit, and patients with stage 4 and 5 CKD showed improvement at 6 months. Our findings align with prior research in animal and cell models, suggesting the potential of EFT to mitigate and potentially reverse progressive kidney function loss in CKD. Compared to traditional herbal formulas based on syndrome differentiation in TCM, EFT may serve as a more suitable adjunctive treatment for CKD in alignment with modern Western medicine. Utilizing GEE for eGFR comparisons across CKD stages in this study indicates that earlier EFT administration for CKD is recommended for optimal renal function protection.

Numerous researchers have identified herbal medicines with anti-inflammatory, anti-oxidative, anti-fibrotic, free radical scavenging, and immune-modulating properties that effectively improve kidney function, reduce the advancement to end-stage renal disease (ESRD), and decrease mortality rates [24]. Commonly used herbal medicines for CKD treatment include *A. membranaceus* [25], *Salvia miltiorrhiza* [26], *Tripterygium wilfordii* [27], *Rheum palmatum* [28], *Panax ginseng* [29], *Coptis chinensis* [30], *Rehmannia glutinosa* [31], *Radix bupleuri* [32], and *Cordyceps sinensis* [33]. According to TCM principles, single herbs are rarely used; instead, complex herbal formulations (comprising two or more herbs) are preferred due to the enhanced medicinal benefits achieved through synergistic interactions between numerous bioactive components [34]. A nationwide population-based study demonstrated that prescribed Chinese herbal medicines, including combination formulas and single Chinese herbal products, reduced the likelihood of ESRD occurrence in individuals diagnosed with CKD [20]. In our prior *in vitro* study, EFT demonstrated enhanced viability and clonogenicity in HK-2 cells (proximal renal tubular cells). Our analysis of apoptosis and fibrosis-related proteins revealed that EFT decreased the expression of indoxyl sulfate (IS)-induced fibrosis-related proteins (α-smooth muscle actin) without impacting apoptosis-related proteins (Caspase 3) **(**not published). In early CKD, inflammation and kidney fibrosis start subtly, with inflammatory cells releasing cytokines and growth factors, leading to initial scarring. Anti-inflammatory, antioxidant, and anti-fibrosis treatments may preserve or partially reverse kidney function at this stage. In late CKD, inflammation becomes chronic and fibrosis worsens, causing extensive scarring and hardening of kidney tissues, ultimately leading to end-stage renal disease where these treatments have limited effects [12]. The progressive fibrosis further diminishes kidney function in a vicious cycle. The varied ingredients in EFT, with strong anti-inflammatory, antioxidant, and anti-fibrosis properties, help mitigate renal function deterioration, especially in early CKD, as shown in our study.

Hypertension often accompanies CKD, acting as a crucial modifiable risk factor that impacts both cardiovascular events and the progression of CKD [35]. Elevated blood pressure exacerbates CKD, regardless of its underlying cause, adversely affecting kidney health [36]. Notably, a significant correlation has been observed between hypertension in CKD and chronic inflammation, with studies highlighting the involvement of inflammatory markers in the progression of both conditions [37]. This inflammation consistently influences pathogenic mechanisms associated with blood pressure and proteinuria in CKD [38]. Our study revealed substantial reductions in systolic blood pressure (SBP) among stage 3B CKD patients after 3 and 6 months of EFT treatment. Furthermore, a decrease in diastolic blood pressure (DBP) was evident among stage 4 CKD patients following three months of EFT therapy. These findings suggest that *C. pilosula* may contribute to modulating blood pressure in EFT-treated CKD 3B patients, other EFT ingredients synergistically contribute to its anti-inflammatory and anti-oxidative effects, working together to collectively reduce blood pressure [39].

Our study showed no notable variations in serum potassium levels among different CKD groups in each subsequent follow-up assessment. There exists a significant correlation between glycated hemoglobin (HbA1c) levels and inflammation [40]. Elevated HbA1c levels correlate with heightened inflammatory markers, particularly high-sensitivity C-reactive protein (hs-CRP). Elevated HbA1c correlates with heightened systemic inflammation, with poorly controlled diabetes mellitus (DM) exacerbating inflammatory activity [40]. This intricate connection between glycemic control and inflammation extends beyond diabetes, impacting conditions like COVID-19, where elevated HbA1c levels are linked to inflammation, hypercoagulability, and adverse outcomes [41]. In our study, individuals with stage 4 CKD experienced a significant reduction in HbA1c without a noticeable change in blood glucose levels after a three-month therapy period. This suggests that EFT ingredients like *A. membranaceus* [42], *C. pilosula* [43], *P. quinquefolius* [44] may contribute to strong anti-inflammatory effects. Despite being composed of five traditional Chinese medicines, EFT demonstrated minimal adverse effects, no increased risk of hyperkalemia, and beneficial effects on hypertension, hyperglycemia, and lipid profile, underscoring its excellent safety profile.

Studies suggest that inflammation can modify the association between LDL cholesterol and outcomes among CKD patients. In CKD, the traditional connection between LDL cholesterol levels and cardiovascular events is modified due to inflammation [45]. This alteration is attributed to the phenomenon of ‘reverse causality,’ where malnutrition and chronic inflammatory conditions contribute to decreased total and LDL cholesterol levels [46]. Despite this, it remains crucial to lower LDL cholesterol to independently reduce cardiovascular risk, emphasizing the significance of managing lipid profiles in CKD patients [47]. In our study, stage 4 CKD patients experienced a substantial reduction in low-density lipoprotein (LDL) levels after 3 and 6 months of EFT treatment. Previous studies indicate that *A. membranaceus* (Huangqi) effectively scavenges superoxide and hydroxyl radicals, with increased activity at higher concentrations. Animal experiments show it significantly reduces plasma total and LDL cholesterol levels while increasing HDL cholesterol levels. *In vivo*, it inhibits free radicals during ischemia-reperfusion, attributing its cardiovascular benefits to potent antioxidant activity [48]. Molecular mechanisms include upregulating HO-1 expression and promoting Akt and Nrf2 phosphorylation, facilitating Nrf2 nuclear translocation to protect vascular endothelial cells from oxidative stress in atherosclerosis treatment [49]. Research suggests that *C. pilosula* helps lower LDL cholesterol levels, likely due to its bioactive compounds regulating lipid metabolism [43]. It also influences disease development through mechanisms of inflammation regulation, oxidative stress, immunomodulation, and apoptosis [50]. However, additional clinical studies are necessary to fully understand and confirm its specific effects on LDL levels and its potential role in managing cholesterol-related disorders.

This study has limitations. Firstly, EFT’s complex composition poses challenges in precisely explaining the therapeutic mechanism for CKD treatment for each ingredient. Additional animal and cellular studies are required to comprehend the molecular signaling pathway of EFT in treating CKD. Secondly, determining the effective timing, optimal dosage regimen, and pharmacokinetics of EFT is crucial for enhancing therapeutic effects and minimizing adverse effects. Thirdly, protein-bound uremic toxins like indoxyl sulfate [51] and increased PTH levels [52, 53] could contribute to sustained low-level inflammation and oxidative stress in CKD, emphasizing the usefulness of measuring IS and PTH levels. Fourthly, a retrospective observational study analyzes existing data to find correlations, while a randomized controlled trial (RCT) assigns participants to groups to establish causality. Retrospective studies generate hypotheses that RCTs can confirm. Thus, our study results should be validated by further RCTs. Future studies should incorporate randomization, placebo control, and follow-up evaluations for a robust conclusion. Mechanism studies, including cellular and animal research, are warranted.

### A narrative literature review of the efficacy of EFT ingredients

**Figure 3:**
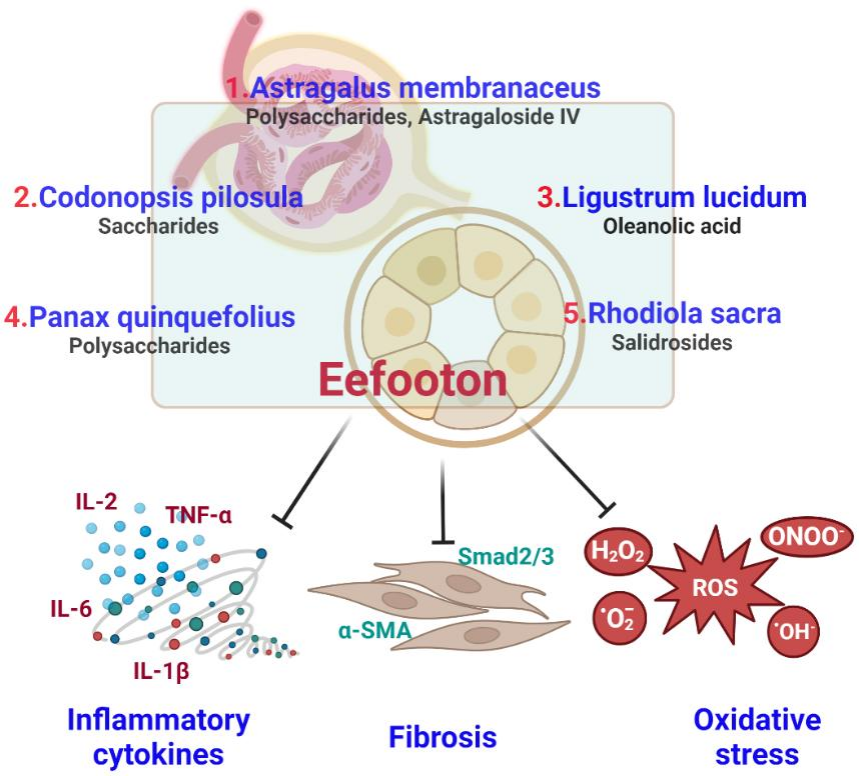
Summary of antioxidant, anti-inflammatory and anti-fibrotic properties of Eefooton ingredients in CKD.

EFT is a liquid blend comprising extracts from five types of herbs, forming an herbal compound designed to address CKD [21, 54]. Table 5 displays the potential signaling pathways associated with each Ingredient included in the EFT formulation, which is used for the therapy of CKD. A summary of the anti-oxidative, anti-inflammatory, and anti-fibrosis properties of EFT are shown in Figure 3.

**Table 5:** Mechanisms of the active ingredients of Eefooton.

**Herbs**	**Active compound**	**Possible mechanisms**
***Astragalus membranaceus***	Astragalus root extract	Increase the effectiveness of conventional therapies by lowering albuminuria, proteinuria, and serum creatinine levels [56].
		Regulation of iNOS activity of macrophages in different states [57].
		Suppression of extracellular matrix deposition and upregulation of VEGF, may reduce capillary loss and improve microstructure dysfunction [75].
		Reduce α-SMA and downregulate E-cadherin; inhibit the induction of EMT and the deposition of extracellular matrix; reduce TGF-β1-induced expression and Smad2/3 phosphorylation [76].
	Polysaccharide Astragaloside IV	Decrease ECM accumulation and inflammatory cell infiltration by inhibiting inflammation via the TLR4/NF-кB signaling pathway [22].
		Inhibit ROS generation and apoptotic protein expression [23].
		Reduce the BUN level and significantly decrease renal oxidative stress [63].
		Decrease albuminuria, s-creat, BUN, ECM expansion, phosphorylation of eukaryotic initiation factor 2α, protein kinase R-like ER kinase and JNK; decrease glucose-regulated protein 78 and 150 kDa oxygen-regulated protein, apoptosis of podocytes, C/EBP homologous protein, and cleaved caspase-3 [67].
		↓ albuminuria, BUN, s-creat; ↓ KiHPCh; ↓ RAS (↓ renin); ↓ MCP-1, TNF-α; ↓ apoptosis; ↑ podocin and nephrin; ↓ ER stress (↓ GRP78, cleaved ATF6, p-PERK, p-IRE1, and CHOP); ↓ ER stress-induced apoptosis (↓ ATF6 and PERK, p-eIF2α, CHOP, p-IRE1α, p-JNK, ↓spliced X-box binding protein 1; ↓ cleaved caspase-12 and caspase-3); ↓ p-mTOR and p70S6 kinase; ↑ p-AMPKα (↑ AMPKα activation); ↑ autophagy; ↑ SERCA2 [68].
		Inhibit the Wnt/β-catenin pathway and reduce the production of EMT-related proteins; lessen oxidative stress injury and the release of inflammatory factors through the interaction of Wnt, PI3K/Akt, NF-κB, Ras, and JAK/STAT signaling pathways [77].
		Decrease the mRNA level of NF-kB and raise the expression of IkB mRNA [78].
		Decrease levels of MDA and 8-OHdG; increase the level of SOD; inhibit oxidative stress and IL-1β and TNF-α overproduction; downregulate ERK1/2 activation and upregulate TRPC6 expression [79].
		Attenuate complement membrane attack complex-induced podocyte injury via the MAPK pathway [80].
		↑SIRT1 → ↓p65 acetylation → ↓NF-κB → ↑ autophagy (↑ Beclin 1 and LC3 II) →↓ MC proliferation and activation; ↓ albuminuria, KiHPCh; ↓α-SMA, FN, and collagen 4 [81].
***Codonopsis pilosula***	Polysaccharides	Inhibit proinflammatory cytokine TNF-α release to decrease renal ischemia–reperfusion injury-induced elevation in serum LDH, AST, BUN, and creatinine levels [82]. Increase glucose uptake and insulin sensitivity in the differentiated adipocytes [83]. Elevate hepatic glycogen and plasma insulin levels [84].
	Oligosaccharides	Improve anti-hypoxia activity by preventing lipid peroxidation and enhancing antioxidant activity [85].
	Selenizing polysaccharide	Promote the phagocytic uptake and NO, TNF-α and IL-6 production; increase IκB-α degradation in the cytosol and the translocation of NF-κB p65 subunit into the nucleus [86].
	Pectic polysaccharide	Promote lymphocyte proliferation; modulate the percentage of CD4+, CD8+, CD28+, and CD152+ T cells; enhance the production of IL-2, TNF-α, and IFN-γ; and increase the expressions of CD28, PI3K, and p38MAPK mRNA [87].
***Ligustrum lucidum***	Oleanolic acid	Modulate glucose levels and regulate lipid metabolism through its hypoglycemic and hypolipidemic properties [88].
		Inhibit cellular inflammatory processes induced by IFN-α of iNOS and of cyclooxygenase 2 in mouse macrophages [89].
		Enhance the proliferative activity of piglet blood lymphocytes and upregulate the CD4 and CD8 cell populations; regulate the expression of Th1- and Th2-related cytokines; elevate the levels of IL-2, IFN-γ, and TNF-α; decrease the levels of IL-4 and IL-10; and stimulate the NO secretion of lymphocytes [90].
	Ligustri lucidi fructus extract	Protective effects against H2O2 toxicity via its free radical scavenging activity and ability to elevate levels of antioxidant enzymes [91].
***Panax quinquefolius***	Polysaccharides	Induce IL-6, IL-1, TNF-α, and IL-10 production in human peripheral blood mononuclear cells [92].
		↑ BW (decreased in T1DM model); ↓ BW, plasma insulin levels, insulin resistance (increased in T2DM model) and ↓ s-glu, HbA1c, albuminuria, s-creat, oxidative stress, HO-1, NF-κB, mesangial expansion, ECM, fibronectin, collagen 4-α1, VEGF, endothelin-1, and TGF-β1 [93].
		Inhibit AGE accumulation in diabetic rat kidneys via their hypoglycemic and renal function ameliorating effects [94].
***Rhodiola sacra***	Salidroside	Decrease the release of inflammatory cytokines and inhibit the TLR4/NF-κB and MAPK signaling pathways [95].
		Reduce MDA levels and elevate glutathione peroxidase activity in a model of kidney damage, induced by unilateral ureter obstruction, through its antioxidant effects [96].
		Reduce cytotoxicity, attenuate ROS accumulation, and decrease intracellular MDA through activation of antioxidant enzymes [97].

8-OHdG, 8-hydroxy-2’-deoxyguanosine; AGE, advanced glycation end-product; AST, aspartate aminotransferase; BUN, blood urea nitrogen; ERK, extracellular signal-regulated protein kinase; HK-2, human kidney 2; ICAM-1, intercellular cell adhesion molecule-1; IFN, interferon; IL, interleukin; iNOS, inducible nitric oxide synthase; LDH, lactate dehydrogenase; MAPK, mitogen-activated protein kinase; MCP-1, monocyte chemoattractant protein-1; MDA, malondialdehyde; mRNA, messenger ribonucleic acid; NF-κB, nuclear factor kappa B; NO, nitric oxide; ROS, reactive oxygen species; SOD, superoxide dismutase; TGF-β1, transforming growth factor beta 1; Th, T helper cells; TLR4, toll-like receptor 4; TNF, tumor necrosis factor; TRPC6, transient receptor potential cation channel, subfamily C, member 6; VEGF, vascular endothelial growth factor.

Huangqi or Astragalus are both common names for *A. membranaceus*, a perennial herb indigenous to northern and eastern China. This herb has been used in TCM for thousands of years and plays a crucial role. The immune system, energy levels, and overall vitality are all promoted through the widespread use of Astragalus in TCM [55]. As a potential therapeutic herb for diabetic nephropathy, *A. membranaceus* has been extensively studied and combined with standard medications can effectively reduce albuminuria, proteinuria, and serum Cr without any observed side effects [56]. Attenuating the induction of the nitric oxide synthase pathway is a potential way to prevent diabetic nephropathy [57]. It demonstrates efficacy in reducing fasting blood glucose, and protinuriaa, reversing glomerular hyperfiltration, and improving early diabetic nephropathy models [58]. *A. membranaceus* is effective against proteinuria in numerous reports [59, 60]. Examining Astragali Radix (the root of the *A. membranaceus*) aqueous extract on rats with Adriamycin (ADR) nephropathy suggests a possible decrease in proteinuria. This is achieved by suppressing the overexpression of endothelial nitric oxide synthase (eNOS) and inhibiting oxidative injury [61]. Clinical research suggests that *A. membranaceus* can stabilize eGFR levels and postpone the initiation of renal replacement therapy in individuals with progressing CKD stage 4. These benefits are linked to the decreased levels of NF-kB [62]. Astragaloside IV (AS-IV), derived from *A. membranaceus*, mitigates oxidative stress, providing renal protection in murine models [63]. It also shows therapeutic promise for cardiovascular disorders [64]. AS-IV has the potential to impede the progression of renal fibrosis by mitigating the TGF-β1/Smad and TLR4/NF-κB signaling pathways, thereby preventing fibrosis [22, 65, 66]. AS-IV also can alleviate endoplasmic reticulum (ER) stress, thereby reducing podocyte apoptosis by suppressing calcium-ATPase type 2 in the sarco-/endoplasmic reticulum (SERCA2) [67, 68].

Animal trials demonstrated that the administration of total flavonoids from Astragalus markedly reduced plasma levels of total cholesterol and LDL while simultaneously increasing HDL levels [48]. Utilizing *Astragalus* polysaccharides led to a decrease in blood levels of fasting plasma glucose, HbA1c, and insulin, coupled with an elevation in superoxide dismutase levels [42]. The antihyperglycemic effect is achieved by increasing levels of glucose transporter protein-4 [69], enhancing PPAR-α activity [70], and inhibiting the NPY (neuropeptide-y) [71]. In a mouse model of iron-deficiency anemia, the *A. membranaceus* polysaccharide-iron (III) complex showed a faster rise in hemoglobin, superoxide dismutase, and catalase levels, along with a quicker decrease in methane dicarboxylic aldehyde levels [72]. *A. membranaceus* also enhanced red blood cell, hemoglobin, and platelet counts in bone marrow cells of mouse models experiencing deficiencies due to myelosuppression induced by irradiation and cytotoxic chemotherapeutic compounds [73].

*Codonopsis pilosula (C. pilosula)*, also known as *Dangshen* or *Codonopsis*, is a perennial flowering plant classified within the *Campanulaceae* family. *Codonopsis* is widely utilized in TCM for its reputed capacity to nurture and strengthen the body, particularly focusing on the spleen and lungs [39]. *C. pilosula* contains assessable bioactive components, including polyacetylenes, phenylpropanoids, alkaloids, triterpenoids, and polysaccharides. These components offer therapeutic benefits similar to Panax ginseng, providing a cost-effective alternative for energy supplementation compared to the relatively pricier Panax ginseng [74]. Scientific evidence supports *C. pilosula’s* role in immune regulation, improved gastrointestinal function, enhanced appetite, lowered blood pressure, and preventive effects against conditions like tumors, diabetes, and aging [39]. *C. pilosula’s* hypoglycemic effects involve reducing oxidative stress, modulating lipid metabolism, enhancing glycolytic enzymes, and lowering liver transaminases. In a type 2 diabetes model, improvements were seen in markers like blood glucose, insulin sensitivity, triglycerides, total cholesterol, LDL/HDL ratio, and malondialdehyde, alongside increased antioxidants such as SOD (superoxide dismutase), TAC (total antioxidant capacity), catalase, and GPX (glutathione peroxidase) [43]. Extracts from the upper parts of *C. pilosula* exhibit stronger antioxidants than their roots. The stems and leaves, rich in active components, hold substantial potential for further research and development [98]. S-CPPA1, a uniform polysaccharide from the stem, offers renoprotective effects against I/R-induced renal injury, possibly by suppressing the release of the pro-inflammatory cytokine TNF-α [82].

*Ligustrum lucidum (L. lucidum),* commonly known as Chinese privet or glossy privet, is an evergreen shrub native to East Asia, particularly China. It has been cultivated for traditional medicine and ornamental horticulture. Often combined with other botanicals, it is used to address health issues related to the liver, kidneys, and immune system [99]. *L. lucidum* contains a variety of chemical elements, including triterpenes, secoiridoids, and flavonoids. The main bioactive constituents are oleanolic acid and ursolic acid [100]. These compounds exhibit various pharmacological effects, providing the plant with hepatoprotective, anticancer, antioxidant, antiviral, anti-osteoporosis, and immunomodulating properties [101]. Moreover, *L. lucidum* demonstrates anti-aging effects, highlighting its versatile therapeutic potential [102].

The ethanol extract of *L. lucidum* fruits (ELL) exhibits mild antioxidant properties. ELL demonstrates a significant reduction in levels of BUN, sGPT, sGOT, alkaline phosphatase, LDH, TG, and Cr at various doses. These findings suggest that ELL, by activating antioxidant enzymes, may protect rats from oxidative damage induced by acute dibutyl hydroxy toluene (BHT) exposure [103]. Fructus Ligustri Lucidi (FLL), extracted from *L. lucidum Ait*. fruit is known for its enduring kidney and liver tonifying properties. Poly pretreatment in UUO mice mitigated glomerulosclerosis and tubulointerstitial fibrosis, reducing key factors (FN, VEGF, MCP-1, Rantes), showcasing FLL’s potential in kidney fibrosis protection [104].

*Panax quinquefolius (P. quinquefolius)*, also known as American ginseng, is a perennial herb belonging to the Araliaceae family. Historically used by Native American communities, it holds significance in traditional medicine systems [105]. Acknowledged for its perceived capacity to enhance energy and alleviate fatigue, *P. quinquefolius* is believed to exert a harmonizing influence on the body’s energy, aligning with the TCM concept of Qi [106, 107]. With its primary bioactive components being diverse plant polysaccharides, *P. quinquefolius* contributes to a spectrum of pharmacological activities. These include immunomodulatory effects [108], antioxidant properties [109, 110], anticancer potential [111], antimicrobial benefits [112], and neuroprotective properties [77]. *P. quinquefolius* exhibits potential to address renal impairment [113]. The main active components in *P. quinquefolius* are dammarane-type ginsenosides, also referred to as saponins. Notably, two distinct variants hold significance: 20(S)-Protopanaxadiol (PPD) and 20(S)-Protopanaxatriol (PPT) [15]. Orally consumed, PPD-type ginsenosides undergo metabolism by gut anaerobes, resulting in the formation of PPD monoglucoside, namely, 20-O-beta-D-glucopyranosyl-20(S)-protopanaxadiol [114]. The key active component in *P. quinquefolius*, *P. quinquefolius* saponin (PQS), effectively inhibits vascular smooth muscle cells (VSMCs) calcification. Its inhibitory effect is associated with decreasing oxidative stress and controlling osteogenic gene expression through promoting Nrf2 upregulation [115]. The AGC1 polysaccharide from *P. quinquefolius* boosts immunostimulatory effects in primary murine splenocytes, leading to increased cellular proliferation, elevated nitric oxide (NO) production, and enhanced tumor necrosis factor-alpha (TNF-α) release [116]. Moreover, extracts of *P. quinquefolius* polysaccharides exhibit the capacity to stimulate the production of IL-6, IL-1, TNF-α, and IL-10 in a controlled laboratory environment [92]. The *P. quinquefolius* root extract effectively lowers blood sugar and HbA1c levels. Additionally, it significantly increases plasma insulin and C-peptide levels in STZ diabetic mouse models [44]. *P. quinquefolius* root extract efficiently reduces blood sugar and HbA1c levels while significantly boosting plasma insulin and C-peptide levels in STZ diabetic mouse models [117].

Ginsenoside Rg1 mitigates sepsis-induced acute kidney injury (AKI) by hindering ferroptosis in renal tubular epithelial cells through the FSP1-CoQ10-NADPH pathway, a ferroptosis suppressor protein 1 (FSP1) mechanism [118, 119]. It also helps prevent the excessive buildup of the extracellular matrix (ECM) in renal tubular cells. Ginsenosides additionally inhibit apoptosis in glomerular mesangial cells and reduce damage to podocytes [120]. Together, these actions suggest ginsenosides as a potential therapeutic strategy for kidney protection, emphasizing their role as a preventive measure rather than a primary medication [121].

*Rhodiola rosea (R. rosea)* is a perennial plant native to mountainous regions in North America, Europe, and Asia. With significant historical importance, it has been widely used in various cultures, especially in the traditional medicine of Siberian and Scandinavian communities [122]. *R. rosea’s* underground components encompass numerous chemical compounds, such as phenols, flavonoids, alkaloids, salidroside, etc. [123, 124]. *R. rosea* is believed to positively affect physical performance and endurance [125]. Previous research indicates cognitive-enhancing properties, potentially improving mental alertness, concentration, and memory [122, 126].

In diabetic kidney disease, salidroside, a bioactive compound in *R. rosea’s*, demonstrates nephron-protective effects by inhibiting apoptosis in proximal renal tubular cells [127]. Modern pharmacological studies show diverse bioactivities in Rhodiola plants, including antioxidant, immunomodulatory, anti-inflammatory, antidiabetic, antihypertensive, neuroprotective, anti-stress, antidepressant, and anticancer properties [128].

Salidroside boosts the expression of erythroid markers, including glycophorin A, transferrin receptor (CD71), and hemoglobin, potentially expediting erythropoiesis in cells treated with erythropoietin [129]. Treating with salidroside improves kidney function, decreases extracellular matrix (ECM) deposition, and mitigates protein levels associated with epithelial-mesenchymal transition (EMT) markers in mouse kidneys and HK-2 cells. Additionally, it markedly reduces the release of inflammatory cytokines and hinders the TLR4/NF-κB and MAPK signaling pathways, indicating Salidroside’s potential as a promising therapeutic approach for renal fibrosis [95]. Salidroside elevates SOD levels in LPS-treated mice by enhancing the expression of Sirtuin 1 (SIRT1) and nuclear factor erythroid 2-related factor 2 (Nrf2) proteins, guarding against LPS-induced kidney injury [130].

## CONCLUSIONS

In this retrospective observational study, EFT improved renal function by increasing eGFR levels and reducing Cr levels alongside conventional CKD treatment. No adverse impact on liver function was noted with EFT treatment. Our analysis of the molecular mechanisms of each EFT ingredient reveals that all five have distinct anti-inflammatory and antioxidant effects. However, *A. membranaceus, L. lucidum, R. rosea* are particularly likely to provide notable antifibrotic effects. The potential improvement in HbA1c levels may be linked to the hypoglycemic effects of *A. membranaceus* and *C. pilosula*. EFT could serve as an adjuvant therapy for CKD due to its potential anti-oxidative, anti-inflammatory, and anti-fibrotic properties. Early administration of EFT in CKD may expedite its protective effects on renal function. However, this observation requires further confirmation.

## MATERIALS AND METHODS

### Patients

This study included patients diagnosed with stable CKD who underwent EFT treatment from March 2019 to March 2021. The 88 participants, ranging from CKD stage 3B to stage 5, comprised 33 women and 55 men aged 30 to 89. Their body mass index (BMI) ranged from 17.35 to 34.41 kg/m^2^. Over 6 months, participants received both conventional medicine and supplementary EFT treatment. The control groups mirrored the EFT cohort in terms of patient count, age (±5%), sex, and eGFR (±5%) for individuals with stable CKD. All patients in the study cohort received conventional medical treatment for CKD. Among the EFT group, 28 patients had diabetes mellitus (DM), and 80 had hypertension. Individuals engaging in self-medication and complementary alternative treatments were excluded from the study.

### Intervention

The Huangqi formula (Eefooton; EFT) utilized in this study comprised *Astragalus membranaceus* (*A. membranaceus*; 3 g), *Codonopsis pilosula* (*C. pilosula*; 3 g), *Ligustrum lucidum* (*L. lucidum*; 3 g), *Panax quinquefolius* (*P. quinquefolius*; 1.3 g), and *Rhodiola sacra (R. sacra*; 1.3 g), diluted in 20 mL of water. Patients were orally administered a 20-mL dose of EFT thrice a day for 6 months, in conjunction with conventional treatment. Conventional treatment encompassed ACE inhibitors, ARBs, or calcium-channel blockers for hypertension; sulfonylureas, dipeptidyl peptidase 4 inhibitors, or insulin for diabetes mellitus (DM); erythropoietin for anemia in the stage 5 CKD group; and statins for dyslipidemia. Notably, EFT holds approval from the United Kingdom Accreditation Service, boasting certifications such as ISO22000 and hazard analysis and critical control points.

### Outcome measurements

The main outcomes assessed in this study were the alterations in eGFR and serum Cr levels. Secondary outcomes encompassed variations in blood pressure, serum potassium, hemoglobin (Hb), glycated hemoglobin (HbA1c), serum aspartate aminotransferase (GOT), and alanine aminotransferase (GPT), and low-density lipoprotein (LDL) cholesterol. These parameters were monitored at three-month intervals throughout the treatment period.

### Statistical analyses

Patient characteristics, encompassing clinic-pathological features, treatment duration, response, age, and sex, were presented as either mean (standard deviation) or count (percentage) based on the variable type. Alterations in laboratory data, such as renal function, liver function, lipid profile, and relevant indicators over the follow-up period, were assessed through generalized estimating equations (GEE). GEE was also employed to compare these changes between EFT-treated patients and controls across distinct CKD stages. The threshold for statistical significance was established at P<0.05. All statistical analyses were conducted using SPSS for Windows, version 22 (Statistics 22, SPSS IBM Corp., Chicago, IL, USA).
